# Relative survival in early-stage cancers in the Netherlands: a population-based study

**DOI:** 10.1186/s13045-020-00888-0

**Published:** 2020-05-12

**Authors:** Avinash G. Dinmohamed, Valery E. P. P. Lemmens, Ignace H. J. T. de Hingh, Otto Visser

**Affiliations:** 1 Department of Research and Development, Netherlands Comprehensive Cancer Organisation (IKNL), Godebaldkwartier 419, 3511 DT Utrecht, The Netherlands; 2 Department of Public Health, Erasmus MC, University Medical Center Rotterdam, Rotterdam, The Netherlands; 3 Department of Hematology, Amsterdam UMC, Cancer Center Amsterdam, Amsterdam, The Netherlands; 4 Department of Surgical Oncology, Catharina Hospital, Eindhoven, The Netherlands; 5 GROW-School for Oncology and Developmental Biology, Maastricht University, Maastricht, The Netherlands; 6 Department of Registration, Netherlands Comprehensive Cancer Organisation (IKNL), Utrecht, The Netherlands

**Keywords:** Cancer, Relative survival, Early-stage, Epidemiology, Registry, Population-based

## Abstract

In this nationwide, population-based study, we assessed 10-year relative survival among 225,305 patients with ten early-stage cancers diagnosed in the Netherlands during 2004–2015. This study aimed to ascertain which early-stage cancer is associated with minimal or no excess mortality and likely to be diagnosed in individuals who are otherwise more healthy or health-conscious than their counterparts in the general population. Ten-year relative survival marginally exceeded 100% in patients with early-stage prostate cancer, while it was close to 100% for patients with ductal carcinoma in situ (DCIS) and stage I cancers of the breast, skin (melanoma), testis, and thyroid. In contrast, patients with early-stage oral/pharyngeal, bladder, lung, and pancreatic cancers experienced considerable excess mortality, reflected by a 10-year relative survival of 74.9%, 69.4%, 45.5%, and 33.9%, respectively. Collectively, the life expectancy of patients with DCIS and early-stage cancers of the prostate, breast, skin (melanoma), testis, and thyroid parallels the expected survival of an age-, sex-, and calendar year-matched group from the general population. Our study findings add to the controversy surrounding overdiagnosis of particular early-stage cancers that are generally not destined to metastasis or cause excess mortality.

To the Editor,

Recently, Marcadis et al. performed a comprehensive analysis of relative survival in 281,970 patients with ten early-stage cancers using data from the Surveillance, Epidemiology, and End Results Program—a study that was hitherto lacking in contemporary literature [1]. They identified five early-stage cancers—i.e., ductal carcinoma in situ (DCIS) and early-stage cancers of the prostate, skin (melanoma), thyroid, and breast—with 10-year relative survival exceeding 100%, suggesting that patients with these early-stage cancers have greater longevity compared to an equivalent group from the general population. This finding favors the premise that these early-stage cancers—which presumably lack metastatic potential and have limited lethal consequences—are generally detected incidentally or via screening in individuals who are otherwise more healthy or health-conscious than their counterparts in the general population [2]. This phenomenon can to some extent be referred to as overdiagnosis [3].

At present, it is unknown whether the findings of Marcadis et al. extend to populations outside the USA. Therefore, we sought to complement their findings by delineating the relative survival of patients with early-stage cancers in the Netherlands.

We selected patients with ten early-stage cancers diagnosed during 2004–2015 from the nationwide Netherlands Cancer Registry (NCR). The selection of early-stage cancers and the study period is congruent with the selection as per Marcadis et al. [1]. The Privacy Review Board of the NCR approved the use of anonymous data for this study.

The primary endpoint was 10-year relative survival, defined as the time from diagnosis until death, emigration, or end of follow-up (February 1, 2019), whichever occurred first. Relative survival was calculated according to the cohort methodology to estimate disease-specific survival [4]. Relative survival is the ratio of the observed survival of patients to the expected survival of an age-, sex-, and calendar year-matched group from the general population. Expected survival was calculated as per the Ederer II methodology using Dutch life tables, stratified by age, sex, and calendar year. All analyses were performed using STATA (version 14.2, StataCorp).

Our analytical cohort included 225,305 patients with ten early-stage cancers diagnosed in the Netherlands during 2004–2015 (Fig. 1). Ten-year relative survival marginally exceeded 100% in patients with early-stage prostate cancer (101.4%; 95% CI, 100.7–102.0%), while it was close to 100% for patients with DCIS and stage I cancers of the skin (melanoma), testis, and thyroid (Fig. 2). Patients with stage I breast cancer experienced minimal excess mortality, while patients with early-stage oral/pharyngeal, bladder, lung, and pancreatic cancers experienced considerable excess mortality (Fig. 2).

**Fig. 1.**
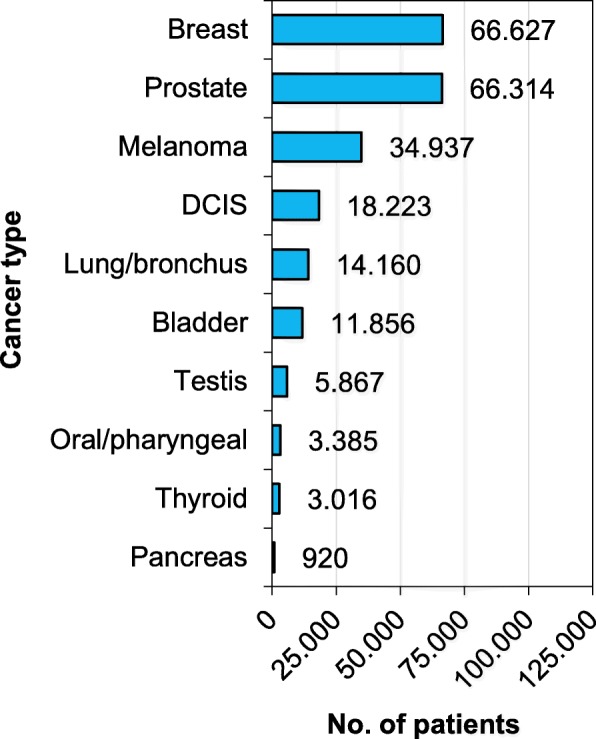
Distribution of early-stage cancers in the Netherlands

**Fig. 2.**
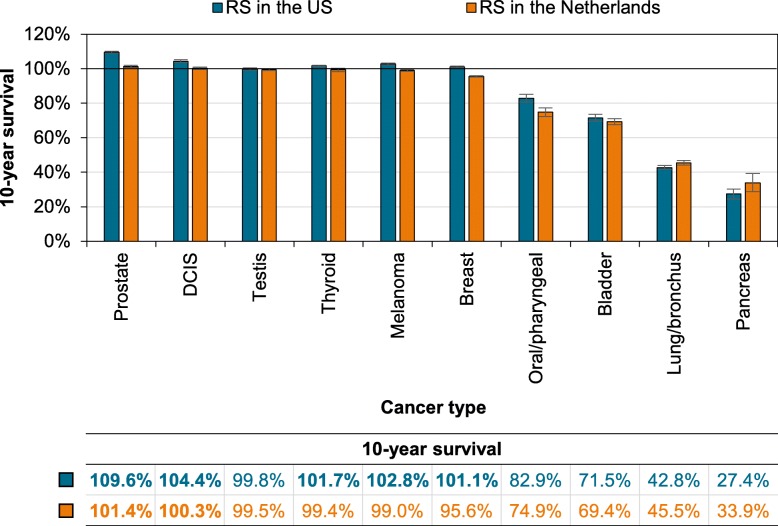
Relative survival of early-stage cancers in the Netherlands compared to the USA. The emerald green bar depicts the 10-year relative survival rate in the USA as per Marcadis et al. [1], the orange bar depicts the 10-year relative survival rate in the Netherlands, and the gray error bar depicts the 95% confidence intervals for the 10-year relative survival rate. The table presents the ten-year relative survival (RS) rates for the ten early-stage cancers according to country. Bold estimates in the table indicate 10-year relative survival rates exceeding 100%. Relative survival is a popular net measure to quantify cancer patient survival with cancer registry data, which measures the survival of cancer patients relative to the expected survival of a comparable group from the general population [4]. Its popularity emanates from the fact that it does not depend on the cause of death, which is often unavailable or unreliable in cancer registry data. In view of this, disease-specific survival—which relies on the accurate classification of the cause of death—might be miscalculated with cancer registry data. Therefore, relative survival is generally considered a proper measure to estimate disease-specific survival and to inform cancer patients about their outlook relative to an equivalent group from the general population. Our study population exclusively includes patients with one primary tumor that has been pathologically confirmed. The stage of the cancer was determined using the Tumor-Node-Metastasis (TNM) classification prevailing at the time of diagnosis. Also, the Gleason scoring system was used to grade prostate cancer. The location and histology of the tumor are registered in the Netherlands Cancer Registry according to the Third Edition of the International Classification of Diseases for Oncology (ICD-O-3)

In this nationwide, population-based study, the life expectancy of patients with early-stage prostate cancer slightly surpassed that of an equivalent group from the general population. This finding is congruent—albeit to a lesser extent—with those observed in the USA (Fig. 2) [1]. Besides, relative survival exceeded 100% among US patients with DCIS and early-stage cancers of the thyroid, skin (melanoma), and breast. In the Netherlands, however, relative survival in these patients ranges between 96% and 100%, which closely mirrors the disease-specific survival in the USA [1] Hence, relative survival in these early-stage cancers is likely to be overestimated in the USA. Seemingly, there is an overrepresentation of health-conscious individuals (e.g., those from higher socioeconomic groups or with adequate insurance coverage) in the US patient population with early-stage cancers. Moreover, these patients have a higher life expectancy than their counterparts in the general population and perhaps more diligently participate in periodic health examinations and cancer screening programs (i.e., healthy-user bias)—all of which may render a cancer diagnosis earlier in the disease course (i.e., lead-time bias) [2, 5, 6]. Sex- and treatment-related factors might also be at play with regard to disparities in relative survival between the Netherlands and the USA. The effect of sex and variation in treatment on relative survival could not be evaluated since information on sex and therapy was not analyzed in the study by Marcadis et al. [1].

Collectively, it can be reasoned that the magnitude of potential cancer overdiagnosis and health inequalities regarding access to screening, diagnosis, treatment, and follow-up services is less extensive in the Netherlands than in the USA, because all Dutch residents have equal access to health care services—regardless of their socioeconomic position and race, ethnicity, and gender. This topic provides an avenue for future research to close the gap in cancer survival between the Netherlands and the USA. This research requires detailed analyses on the drivers of survival disparities in early-stage cancers between the Netherlands and the USA.

## Data Availability

The data that support the findings of this study are available via The Netherlands Comprehensive Cancer Organisation. These data are not publicly available and restrictions apply to the availability of the data used for the current study. However, these data are available upon reasonable request and with permission of The Netherlands Comprehensive Cancer Organisation.

## Abbreviations

CI: Confidence interval
DCIS: Ductal carcinoma in situ
US: United States
NCR: Netherlands Cancer Registry
